# The relationship between muscle oxygen saturation kinetics and maximal blood lactate accumulation rate across varying sprint cycle durations

**DOI:** 10.1002/ejsc.12242

**Published:** 2025-02-27

**Authors:** Michael Porter, Jamie Langley

**Affiliations:** ^1^ Centre for Physical Activity, and Life Sciences University of Northampton Northampton UK; ^2^ Department of Higher Education Sport Loughborough College Loughborough UK

**Keywords:** maximum lactate accumulation rate, MOXY, muscle oxygen, NIRS, sprint cycling

## Abstract

This study evaluated the relationship between muscle oxygen saturation (SmO_2_) and the maximal blood lactate accumulation rate (vLa_max_) during three test durations (10, 15 and 30 s) to validate the optimal test duration of vLa_max_ protocol. Thirteen developmental trained males (age: 27 ± 6 years and peak power: 1133 ± 185W and 14.88 ± 1.61 W·kg^−1^) performed three maximal cycle tests (10, 15 and 30 s). Performance metrics were measured throughout; peak power, mean power, and cadence. vLa_max_ was determined using blood lactate concentrations following each test. SmO_2_ of the vastus lateralis was measured using a MOXY device via near‐infrared spectroscopy, throughout all experimental conditions. The shortest test (10 s) produced a significantly (*p* = 0.005; *p* < 0.001) higher vLa_max_ (0.83 ± 0.15 mmol·L^−1^·s^−1^) than 15 s (0.67 ± 0.13 mmol·L^−1^·s^−1^) and 30 s (0.43 ± 0.06 mmol·L^−1^·s^−1^). Three relationships between SmO_2_ kinetics and vLa_max_ were observed: (1) a very strong inverse relationship (*r* = −0.994, *p* < 0.001) between SmO_2_ desaturation and vLa_max_ time dependent kinetics, (2) a significant inverse relationship (*r* = −0.648, *p* < 0.001) between SmO_2_ time spent at the nadir and vLa_max_ and (3) a moderate relationship (*r* = 0.508, *p* = 0.11) and similar time to attain the SmO_2_ nadir (8.47 ± 1.50s) and vLa_max_ (8.92 ± 0.77s). These results validate the 10‐s test duration for determination of vLa_max_ verified with mathematical modelling predicting peak vLa_max_ occurs at ∼9 s. SmO_2_ desaturation closely reflects the vLa_max_ kinetics, with the time points of the SmO_2_ nadir and peak vLa_max_ closely corresponding.

## Highlights

- The vLa_max_ training principle is gaining traction in applied sports science; however, the optimal test duration and energy determinants are still unknown and reliant on mathematical modelling.
- Our results demonstrate that the shortest test duration of 10 s elicited the highest vLa_max_ with mathematical modelling predicting vLa_max_ peaks at ∼9 s during all‐out sprint cycling.
- Using mNIRS to monitor SmO_2_ desaturation kinetics closely reflect the vLa_max_ kinetics, with the time points of the SmO_2_ nadir and peak vLa_max_ closely corresponding.
- The use of mNIRS to monitor an individual's SmO_2_ desaturation profile during all‐out sprint cycling may provide vital practical applications in determining the optimal vLa_max_ test duration.

**Abbreviations**
ATP: Adenosine triphosphate
α: Alpha
BM: Body mass
β: Beta
BLa: Blood lactate concentration
*d*
: Cohen’s d
*n*
: Sample size
*η* ^*2*^_*p*_
: Partial eta squared
NIRS: Near‐infrared spectroscopy
mNIRS: Muscle Near‐infrared spectroscopy
PCr: Phosphocreatine
rpm: Revolutions per minute
SmO_2_
: Muscle oxygen saturation
*T*
: Time (minutes)
tHb: Tissue hæmo(+myo)globin saturation
τ_FC_
: Fast component time constant
τ_SC_
: Slow component time constant
t½: Half‐time
vLa_max_
: Maximal lactate accumulation rate
V̇O_2Base_
: Baseline oxygen uptake prior to test procedures
Δ: Change

## 1 INTRODUCTION

The non‐invasive identification of the in exercise contribution from all three energy systems (phosphagen, glycolytic, and oxidative) is widely sought after within exercise physiology. Currently, due to the rapid fluxes within the percentage contribution of each energy system during exercise, there is no non‐invasive gold standard measure of the glycolytic energy contribution (Yang et al., 2023). The oxidative component of exercise has been widely established via the use of cardiometabolic carts, utilising expired V̇O_2_/V̇CO_2_ to determine substrate utilisation, and total oxidative energy expenditure (Van Hooren et al., 2023). Likewise, the recovery portion of the tissue oxygen saturation (SmO_2_) via near‐infrared spectroscopy (NIRS), has been considered a proxy of resynthesis of phosphocreatine (PCr) (McCully et al., 1994). Recently, the maximal lactate accumulation rate (vLa_max_) protocol has gained traction as a non‐invasive method to determine the glycolytic contribution of exercise (Adam et al., 2015; Langley et al., 2024; Yang et al., 2023).

NIRS provides a non‐invasive assessment of SmO_2_ and tissue haemodynamic status (Boushel & Piantadosi, 2000; Ferrari & Quaresima, 2011). NIRS additionally provides real‐time visualisation of SmO_2_ of the working peripheral muscles, most commonly the *vastus lateralis* (Perry et al., 2024). Prominent changes in desaturation, (re)saturation profiles, and recovery time course of SmO_2_ has been strongly correlated to PCr resynthesis (Buchheit & Ufland, 2011; McCully et al., 1994). The resynthesis of PCr levels following high intensity exercise is dependent on the production of adenosine triphosphate (ATP) by the mitochondria, which is reliant on the availability of oxygen in the myoglobin (Dunst et al., 2023). Oxygen supply and availability during periods of desaturation has been strongly correlated to PCr dephosphorylation (Hamaoka et al., 1996). Additionally, periods of fast SmO_2_ desaturation have been associated with a greater energy demand from the glycolytic system (Boushel et al., 1998). However, the exact relationship between the PCr and glycolytic system using NIRS has not yet been established. The speed of the SmO_2_ desaturation and resultant PCr dephosphorylation could be strongly affected by the glycolytic flux of energy production (Dunst et al., 2023).

Following maximal exercise, namely cycling sprints, PCr is rephosphorylated by creatine kinase, where ATP is produced by mitochondrial oxidative phosphorylation, and glycolysis. Following the onset of maximal exercise, glycolytic flux rapidly increases associated with elevated lactate production (Jacobs et al., 1983). It has been hypothesised that oxidation of lactate during exercise is attributed to maintaining energy equilibrium, and is reflected by SmO_2_ kinetics. Therefore, peak SmO_2_ desaturation reflect PCr kinetics and correlate with vLa_max_ (Dunst et al., 2023).

Since the inception of the vLa_max_ protocol, the academic community has questioned its validity at determining the glycolytic metabolic contribution. Therefore, this study aims to identify if the vLa_max_ protocol could further validate the inclusion of SmO_2_ to identify the energy system contributions (Beneke et al., 2002; Heck et al., 2003). Recently, the vLa_max_ literature has identified a 10‐s test duration is significant at eliciting the highest values and may mitigate the oxidative component of ATP resynthesis, when compared with 15‐ and 30‐s test durations (Langley et al., 2024). Therefore, this study has three key aims: (1) Validate the optimal vLa_max_ test duration using a proxy of SmO_2_. (2) Identify the correlation of the SmO_2_ and vLa_max_ kinetics. (3) Establish if NIRS can be used to identify PCr dephosphorylation time constant for the three test durations. It is hypothesised that the application of mNIRS to monitor SmO_2_ kinetics will associate with the time dependent vLa.

## 2 MATERIALS AND METHODS

### 2.1 Participants

Thirteen developmental level males participated in this study (Table 1) (McKay et al., 2021). All participants were habituated to maximal sprint cycling prior to the study. Ethical approval was granted by the Loughborough College Ethics Committee in accordance with the Declaration of Helsinki. Prior to testing, participants provided written informed consent and completed a PAR‐Q form confirming they were free from illness, disease or injury.

**TABLE 1:** Participant characteristics (*n* = 13).

Participant characteristics	
Variable	Mean ± standard deviation
Age (years)	27 ± 6
Mass (kg)	76.6 ± 12.4
Stature (cm)	178 ± 9
Thigh skinfold thickness (mm)	8.8 ± 2.7
Femur length (cm)	44.0 ± 3.4
Peak power^a^ (W)	1133 ± 185
Peak power^a^ (W·kg^−1^)	14.88 ± 1.61

^a^ Peak power assessed via a *v*La_max_ protocol.

### 2.2 Study design

The data presented here were collected as part of a previously published research (Langley et al., 2024). This study was a single‐blind study, with repeated measures design with three counterbalanced experimental conditions: 10, 15 and 30 s. Participants were randomly assigned an experimental order and blinded to the order (sprint duration) until the day of each trial, to minimise order effect. Prior to any experimental trials participants were familiarised with the hardest physiological trial (30 s). All experimental trials were separated by a minimum of 48 h but a maximum of 96 h to minimise peripheral muscle fatigue (Ide et al., 2011). Participants conducted all trials at the same time of day ± 2‐h over a 14‐day period. To minimise the influence of carbohydrate ingestion, participants only consumed water within the 2 h prior to each testing session (Adam et al., 2015). Participants abstained from consuming caffeine within 4 h of each test (Brachtel & Richter, 1992).

### 2.3 Experimental protocol

To test if SmO_2_ can validate an optimal vLa_max_ test duration, participants performed three sprint cycle tests of varying durations in line with established literature (Adam et al., 2015; Hommel et al., 2019). Prior to the familiarisation session, participants' stature (cm), mass (kg), and thigh skinfold (mm) were established. Participants subsequently undertook a warm‐up comprising of 12‐min cycling at a power output of 1.5 W·kg^−1^ on a static cycle ergometer (Excalibur, Lode, Groningen, Netherlands). A 5‐s sprint was conducted at a power output of 7.5 W·kg^−1^ after the initial 6 min to prime the physiological systems for the subsequent maximal cycle test (Tomaras & MacIntosh, 2011). Following 12‐min warm‐up, participants performed an active recovery, cycling at 50 W for 10 min to aid a reduction of hyperlactataemia. During the last two minutes of the active recovery, the right hand was perfused in 39 ± 1°C water for 120 s to establish enhanced hyperaemia prior to lactate sampling (An et al., 2019). Two concurrent capillary blood samples (20 μL) from the right index finger were collected following 60‐s post‐active recovery to establish pre‐exercise blood lactate (BLa, mmol·L^−1^) concentrations (BLa_pre_), and intra‐reliability of sampling (Hommel et al., 2019). All capillary blood samples were analysed using a dual channel Biosen C‐line lactate analyser (EKF‐diagnostic, Barleben, Germany).

Precisely 120 s following the active recovery, participants performed either the 10, 15 or 30 s (t_test_) all‐out sprint test on a Wattbike Pro Model B dependent on the randomised order. The sprint test was performed with the air brake resistance set to level 10, and the magnetic resistance set at level 1 (Herbert et al., 2015; Porter et al., 2022). The sprint test was performed from a static seated position with the participants’ left leg at 140° from the bottom of the pedal stroke to minimise muscular occlusion in NIRS (Abbiss et al., 2019). Participants were instructed to pedal maximally, with loud vocal motivation provided throughout.

Peak and mean power output (W and W·kg^−1^), and cadence (rpm), were sampled throughout at 100 Hz, with angular velocity calculated twice per pedal revolution, and fatigue index (FI%) calculated (Equation (1)). Power data were averaged per pedal revolution for statistical analysis. At the cessation of the test, capillary BLa samples were collected every 60 s for 15 min to identify the maximum measured BLa concentration (BLa_maxpost_). Expired air was collected using the Cortex Metalyser 3B, calibrated with known quantities of gases and the flow rate via a 3‐L syringe prior to each test undertaken. Exercise respiratory gases (V̇O_2_ and V̇CO_2_) were sampled throughout testing and the following 15‐min post exercise. Following the post‐exercise sample period, an instructed 5‐min self‐paced cool down was conducted on a Wattbike.

(1) $$\text{Fatigue}\;\text{Index}=\frac{\left(Peak\;Power\;\left[W\right]-Lowest\;Power\;\left[W\right]\right)}{Peak\;Power\;\left(W\right)}\times 100$$

The fatigue index was calculated using lowest power output (W) and peak power output (W) (Adams & Williams, 2002).

### 2.4 *v*La_max_ calculations

The *v*La_max_ was derived from BLa_pre_, BLa_maxpost_, the alactic time interval (t_alac_) and test duration (t_test_) using Equation (3) (Mader, 1994). The period from the onset of the sprint to physiological decline in peak power output by greater than 3.5% establishes the end of the alactic time interval (Hauser et al., 2014). The time to attain peak *v*La_max_ was modelled using a biexponential model (Equation (4)). *v*La_max_ is the calculated maximum of the interpolated *v*La(t) (mmol·L^−1^·s^−1^) at a given time point; *t* denotes time (s); *v*La_rest_ is 0 where it is assumed no lactate is produced during the alactic time period and the time delay (TD) denotes when the alactic time period ends and *v*La production begins. A denotes the extravascular increase, and k_1_ and k_2_ the rate constant increase and removal in *v*La, respectively. The starting values for A, k_1_ and k_2_ were the Δ rest to *v*La_max_, 0.3 and 0.05, respectively.

The phosphagen energy contribution during each sprint test were established using EPOC determined by non‐linear regression using a biexponential 4‐parameter model in accordance with Knuttgen (1970). A_FC_ and A_SC_ represent the amplitudes of the fast and slow components, respectively, τ_FC_ and τ_SC_ the corresponding time constants and V̇O_2Base_ the asymptotic resting oxygen uptake; the TD is ustilised to compensate for any delay in the measurement (Equation (2)). The time duration of EPOC was determined by the period of post‐exercise respiratory gases returning to pre sprint test ‘baseline’ values.

(2) $$\dot{V}O_{2}\;\text{EPOC}\;\left(t\right)=\text{Afc}\cdot e+\text{Asc}\cdot e+{\text{VO}}_{2\text{Base}}$$

*EPOC calculation* Fc = fast component, sc = slow component and A = amplitude.

The total PCr work achieved following the 30‐s sprint was defined as 100% PCr work and respective percentages of PCr work following the 10‐ and 15‐s sprints calculated from this value used to model PCr work overtime. The replenishment of high‐energy phosphates (V̇O_2_PCr and V̇O_2_FC) was estimated by the product of the amplitude (AFC) and time constant (τFC) of the fast component. PCr work (J) was estimated by applying a calorific equivalent of 20.9 J.ml^−1^ to the volume corresponding with the V̇O2FC (Di Prampero, 1981).

(3) $$v\;{\text{La}}_{\max}=\frac{BLa\;maxpost-BLa\;pre}{Ttest-Talac}$$

*vLa*_*max*_ *calculation* (Mader, 1994) BLa_maxpost_ = maximum post‐exercise blood lactate, BLa_pre_ = blood lactate prior to the start of the test, t_test_ = test duration, t_alac_ = alactic time interval and *v*La_max_ = maximal lactate accumulation rate.

(4) $$v\;{\text{La}}_{\max}\;\left(t\right)=v{\text{La}}_{\text{rest}}+A\;\cdot \;\left(1-e{-}^{k1\cdot t-\text{TD}}\right)-A\;\cdot \;\left(1-e{-}^{k2\cdot t}\right)$$

Time course of *v*La_max_ kinetics was modified from Dunst et al., 2023.

**FIGURE 1:**
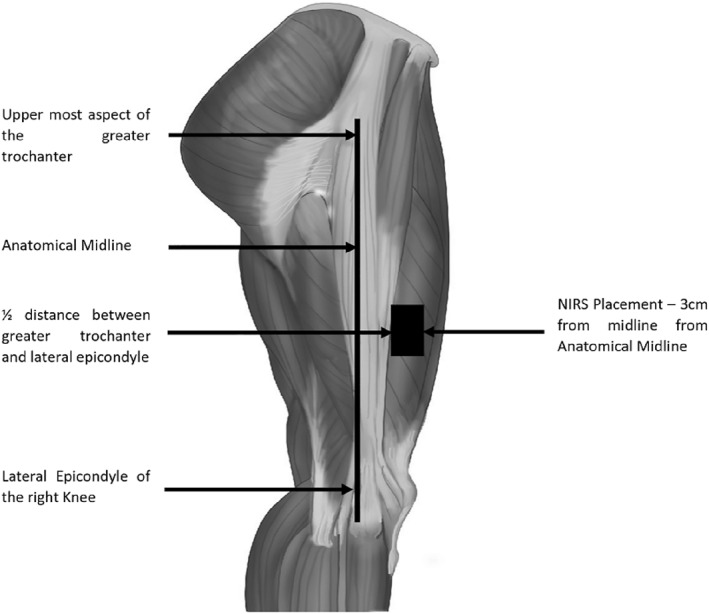
MOXY placement on the *vastus lateralis*.

### 2.5 Near‐infrared spectroscopy

To measure peripheral SmO_2_ status, participants were required to wear a small NIRS device affixed to the muscle belly of the *vastus lateralis* muscle (Porter et al., 2022) (Figure 1) (MOXY, Fortiori Design LLC, Minnesota, USA). Prior to attachment, participants’ bodily hair was removed within placement area (10 × 10 cm) and sterilised. Device placement location was marked with indelible ink to ensure accurate repeat placement. The device was placed in a flexible polyurethane skirt that fits around the Moxy sensor to prevent ambient light travelling through the tissue, interfering with the measurement. The device and the skirt were affixed to the muscle belly via a pre‐cut adhesive tape (BSN Medical Ltd., Hull, UK). Participants were required to wear long Lycra shorts to add additional ‘black out’ effects to the device. A skinfold thickness (Harpenden Skinfold Caliper, Baty International, West Sussex, England) of the thigh was measured to assess peripheral adiposity levels. A maximum skinfold thickness of 12.5 mm was set to ensure that penetrative depth of the MOXY device (12.5 mm) was greater than the adipose tissue.

**FIGURE 2:**
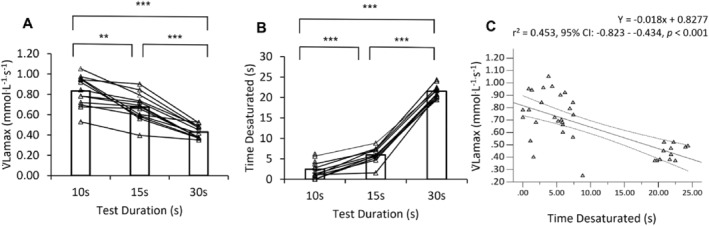
A three‐panel plot illustrating the relationship between vLa_max_ and time spent desaturated. A, Comparisons of *v*La_max_ between different exercise durations (10, 15 and 30s) of all‐out cycling; bars denote the group mean, and triangles illustrate individual participants. B, Comparisons between the time spent desaturated and exercise duration; bars denote the group mean and triangles illustrate individual participants. C, The relationship between *v*La_max_ and the time spent desaturated. Triangles denote individual data points, the solid black line illustrates linear trend line, and dashed lines illustrate 95% confidence intervals. *Asterisks denote statistically significant difference in v*La_max_ : *p < 0.05, **p < 0.01 and ***p < *0.001.*

The MOXY device expresses muscle tissue oxygen saturation (SmO_2_) and changes in total tissue hæmo(+myo)globin (tHb). Data were acquired at 2 Hz from the sensors’ internal memory and was digitally downloaded following each test. SmO_2_ data were split into three distinct classifications: reoxygenation rate, deoxygenation rate and change (Δ). The deoxygenation rate (deoxy rate %·s^−1^) was calculated as the ΔSmO_2_ (%) from the onset of desaturation to the nadir of SmO_2_ by fitting a 10‐s linear model to the time component of the SmO_2_ (%) decline. The deoxygenation rate was analysed over the first 10 s to allow comparisons between exercise bouts. Likewise, the reoxygenation rate (reoxy rate %·s^−1^) was calculated as the ΔSmO_2_ (%) from the onset of the resaturation (greater than 2% per second) by fitting a linear model to the mean of the time constants 18 s part of the SmO_2_ (%) recovery. The slope of the relationship was retained as an index of the reoxygenation rate.

Additionally, non‐linear regression models were applied to SmO_2_ (%) kinetics to determine the primary component time constant (τ) and half‐time (t½) for both the re(de)saturation kinetics. Both re(de)saturation non‐linear models utilised a three‐parameter monoexponential equation and fitted using least sum of residuals squared function (Equation (5) and Equation (6)). The recovery of SmO_2_ (%) applied a TD function and fit to the first 60 s of resaturation (Buchheit & Ufland, 2011). If the fit of the monoexponential model was *r* ^2^ < 0.90, data were excluded from analysis. The amplitude (A) was calculated from the baseline to the nadir and from nadir to peak recovery SmO_2_ (maximum 2 s average). SmO_2_ data were processed using the methodology suggested in Rodriguez et al. (2018). A two‐second moving average filter was applied to attenuate the ‘noise’ in the NIRS signal, while retaining the integrity of the original data.

(5) SmO2(t)=SmO2lowest+A2·[1−e−((t–TD·k3))^]

The above equation shows recovery rate monoexponential calculation where SmO_2_ lowest is the lowest value prior to a sustained (re)saturation, A_2_ is the amplitude, k_3_ denotes the constant rate of increase and TD is the time delay (modified Buchheit et al., 2011).

(6) SmO2(t)=SmO2baseline–A3·[1−e−((t·k4))^]

The above equation shows desaturation rate monoexponential calculation where the SmO_2_ baseline is the 30‐s average (SmO_2_%) before each test (modified Buchheit et al., 2011).

### 2.6 Statistical analysis

Statistical analysis was performed using SPSS Statistics version 29.0 (SPSS, IBM Corp software, IL, USA). A priori power analysis revealed 15 participants provide significant power to detect significant differences at an α‐level of 0.05 (β‐level of 0.8) for the primary outcome (SmO_2_, [G*POWER 3.1 software, Dusseldorf, Germany]). Two participants were excluded due to skinfold thickness exceeding 12.5 mm; thus, a sample size of 13 was used for data analysis. The α‐level of *p* = 0.05 was applied for all analyses. Normality of all data was assessed using the Shapiro–Wilke test.

A one‐way repeated measure analysis of variance (ANOVA) was used to assess differences between the three conditions (10, 15 and 30 s) for *v*La_max_, ΔSmO_2_ (%), and deoxy rate (%·s^−1^) and reoxy rate (%·s^−1^), respectively. Effect size was calculated using partial eta squared (*η* ^*2*^_*p*_) (small 0.01–0.05, medium 0.06–0.13 and large ≥0.14) (Bakeman, 2005). Post hoc Bonferroni corrections were applied, using Cohen's d to estimate effect sizes (small 0.2–0.49, medium 0.5–0.79 and large ≥0.8).

Relationships between mNIRS variables (re)saturation rate, desaturation rate (%·s), amplitude of desaturation (ΔSmO_2_%) and *v*La_max_ were analysed using Pearson's correlation coefficient across each trial duration, 95% CI applied Pearson correlation bias adjustment. The relationship between *v*La_max_ and SmO_2_ time spent at the nadir was analysed using Spearman's correlation with 95% CI estimates corrected using Fieller’s, Hartley’s and Pearson’s adjustments (negligible 0.00–0.09, weak 0.01–0.39, moderate 0.40–0.69, strong 0.70–0.89 and very strong 0.90–1.00).

## 3 RESULTS

The intraclass correlation coefficient (ICC) was calculated using a single measurement, two‐way mixed model using absolute agreement for peak power (W), maximum revolutions per minute (rpm) and ΔSmO_2_ (%) (poor <0.5, moderate 0.51–0.75, good 0.76–0.89 and excellent >0.9) (Koo & Li, 2016). Excellent ICC was observed for both peak power (W) (0.961; 95% CI: 0.893 and 0.987) and maximum rpm (0.921; 95% CI: 0.763 and 0.975). Moderate ICC was observed for ΔSmO_2_ (%) (0.688; 95% CI: 0.122 and 0.915).

### 3.1 Relationship between *v*La_max_ and mNIRS SmO_2_ kinetics

Comparisons of the time spent desaturated (lowest SmO_2_%) across the three all‐out sprint cycle durations were analysed via a one‐way repeated measures ANOVA. Results demonstrated a significant difference (*p* < 0.001) between groups, associated with a large effect size (*η* ^*2*^_*p*_ = 0.988). The 10‐s test duration time spent desaturated was significantly shorter (*p* < 0.001 and *d*: 2.17) than the 15‐s and the 30‐s test durations (*p* < 0.001 and *d:* 11.82). Additionally, the time spent desaturated for the 15‐s test duration was significantly shorter (*p* < 0.001 and *d*: 8.79) than the 30‐s test duration (Table 2). Figure 2 illustrates the moderate significant inverse relationship observed between *v*La_max_ and the time spent desaturated (*r* = −0.673, *r* ^2^ = 0.453, 95% CI: −0.823 to −0.0434 and *p* < 0.001). No significant correlations were observed between the ΔSmO_2_ (%) desaturation and the *ν*La_max_ across test durations (10 s: *r* = 0.311, *r* ^2^ = 0.097, 95% CI: −0.302–0.730 and *p* = 0.301; 15 s: *r* = 0.485, *r* ^2^ = 0.0235, 95% CI: −0.234–0.846 and *p* = 0.155; 30 s: *r* = 0.479, *r* ^2^ = 0.229, 95% CI: −0.117–0.808 and *p* = 0.097).

**TABLE 2:** Comparison in muscle oxygenation variables and *v*La_max_ across three experimental conditions (10, 15 and 30s) *n* = 13.

Exercise duration	10 s	15 s	30 s		
mNIRS variables	M ± SD	M ± SD	M ± SD	*p*‐value	*η* ^*2*^_*p*_
SmO_2_ lowest (%)	15.10 ± 22.94	18.90 ± 20.60	14.20 ± 12.69	*p* = 0.76	*η* ^*2*^_*p*_ = 0.03
SmO_2_ max (%)	82.70 ± 5.03	80.30 ± 8.74	83.30 ± 6.48	*p* = 0.53	*η* ^*2*^_*p*_ = 0.07
ΔSmO_2_ (%)	67.60 ± 20.49	61.4 ± 15.21	69.20 ± 10.48	*p* = 0.33	*η* ^*2*^_*p*_ = 0.12
Max SmO_2_ desaturation rate (%·s)	7.27 ± 2.00	9.89 ± 4.12	13.68 ± 4.44	*^a^* *p* < 0.01	*η* ^*2*^_*p*_ = 0.57
Max SmO_2_ (re)saturation rate (%·s)	4.09 ± 1.86	4.20 ± 1.37	5.50 ± 1.95	*p* = 0.14	*η* ^*2*^_*p*_ = 0.20
Time to desaturate (s)	8.17 ± 1.79	9.07 ± 1.97	8.81 ± 1.47	*p* = 0.24	*η* ^*2*^_*p*_ = 0.15
Time to (re)saturate (s)	58.18 ± 28.77	56.58 ± 24.94	55.58 ± 24.60	*p* = 0.97	*η* ^*2*^_*p*_ < 0.01
^1/2^ *t* to (re)saturate (s)	10.29 ± 5.48	9.89 ± 4.53	9.47 ± 4.03	*p* = 0.91	*η* ^*2*^_*p*_ = 0.01
Mean response time to (re)saturate (s)	14.55 ± 7.20	14.14 ± 6.23	13.90 ± 6.15	*p* = 0.97	*η* ^*2*^_*p*_ < 0.01
K_3_ SmO_2_ resaturation constant	0.07 ± 0.03	0.07 ± 0.03	0.07 ± 0.04	*p* = 0.88	*η* ^*2*^_*p*_ < 0.01
K_4_ SmO_2_ desaturation constant	0.25 ± 0.06	0.21 ± 0.05	0.29 ± 0.08	*p* = 0.83	*η* ^*2*^_*p*_ < 0.29
Time spent desaturated (s)	1.84 ± 1.79	5.93 ± 1.97	21.20 ± 1.47	*^a^* *p* < 0.01	*η* ^*2*^_*p*_ = 0.99

^a^ Denotes significance *p* < 0.05; SmO_2_: muscle oxygen saturation; m: mean; SD: standard deviation; η^*2*^_*p*_: partial eta squared; Δ: Delta and *t:* time.

**FIGURE 3:**
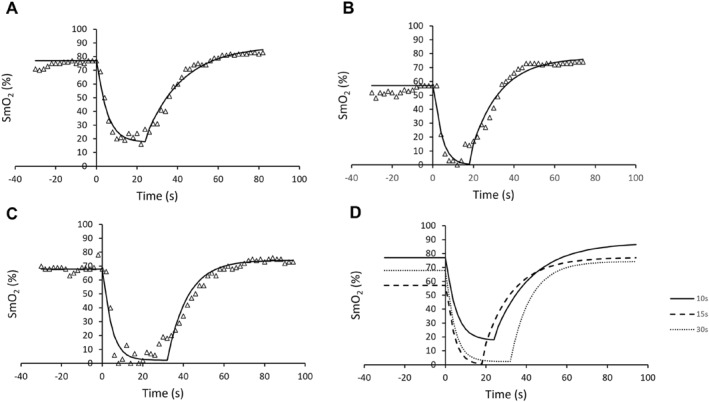
Example SmO_2_ data model fit for participant number four. Triangles denote individual data points, and solid black lines illustrate the model fit applied for data analysis. All‐out sprint cycling starts at 0 s, with the average SmO_2_ for the 10 s prior established as the baseline. A monoexponential decay was fit for the SmO_2_ desaturation and resaturation was modelled using a monoexponential growth factor over a 60‐s duration. SmO_2_ responses following 10s (A), 15s (B) and 30s (C) all‐out sprints are illustrated; all three SmO_2_ responses are combined in figure D.

A significant difference (*p* = 0.01) was observed for the maximum desaturation rate (%·s) across the three test durations, associated with a large effect size (η^2^_p_ > 0.57). During the 10‐s test duration, the maximum desaturation rate was significantly slower (*p* = 0.007 and *d*: 1.86) than the 30‐s, but not the 15‐s test duration (Table 2). An example analysis of SmO_2_ kinetics is illustrated in Figure 3; the model fit for both de(re)saturations was *r* ^2^ ≥ 0.90 for all participants across all test durations (resaturation model fit: *r* ^2^ = 0.960 ± 0.034 and desaturation model fit: *r* ^2^ = 0.940 ± 0.029). No other significant differences were observed between remaining SmO_2_ kinetic variables (Table 2).

**FIGURE 4:**
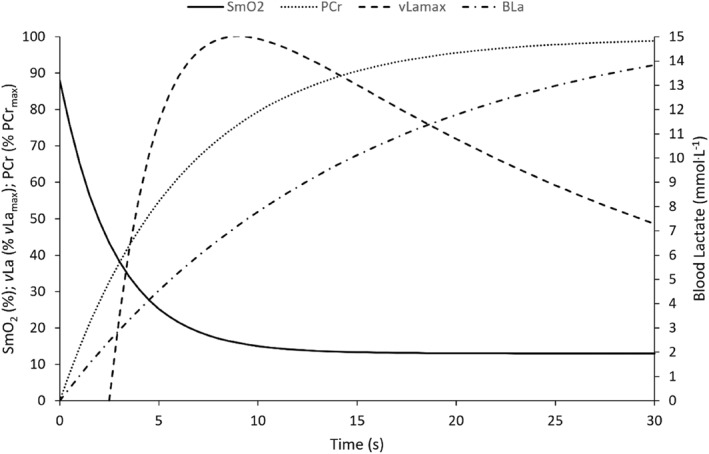
Mean time‐dependent O_2_ saturation SmO_2_ (t) [%] of the vastus lateralis, time‐dependent PCr energy contribution (%PCr_max_), time‐dependent *v*La_max_ (% *v*La_max_) and time‐dependent blood lactate kinetics (mmol·L^−1^) during 30‐s all‐out cycling test.

### 3.2 Time course of the *v*La_max_ and energy system interaction

Figure 3, illustrates a very strong negative correlation (*r* = −0.994, *r* ^2^ = 0.988, 95% CI: −0.999 to −0.964 and *p* < 0.001) between the SmO_2_ (%) kinetics of the vastus lateralis and the *v*La_max_ (%) kinetics analysed from the end of the alactic time interval (2.5 s) to the time peak *v*La_max_ was attained (10 s). The mean time to peak *v*La_max_ (8.92 ± 0.77 s) closely reflects the SmO_2_ time duration to the nadir (8.47 ± 1.50 s), associated with a moderate correlation (*r* = 0.508, *r* ^2^ = 0.336, 95% CI: −0.157–0.842 and *p* = 0.11). A perfect negative correlation (*r* = −1.0, *r* ^2^ = 1.0 and *p* < 0.001) was observed between the time dependent desaturation of SmO_2_ (%) and PCr work. While a close time interval between the SmO_2_ desaturation ^1/2^ *t* (3.04 ± 1.21 s) and the alactic time (2.52 ± 0.99 s) was observed, there was no significant correlation (*r* = 0.130, *r* ^2^ = 0.0169 and 95% CI: −0.228 to −0.457, *p* < 0.464) (See figure 4).

### 3.3 Performance data and *v*La_max_ interactions across exercise duration

All performance variables (peak power, fatigue index, max rpm and mean power) were demonstrated to have a significant difference across the three‐test duration (*p* < 0.05, Table 3). A strong significant correlation (*r* = 0.793, *p* < 0.001, 95% CI: 0.636–0.886 and *n* = 39) was observed between *v*La_max_ and mean relative power (W·kg^−1^). Mean power (W·kg^−1^) during the 30‐s test duration was most strongly correlated with *v*La_max_ (*r* = 0.755, *p* < 0.003 and 95% CI: 0.322–0.918) compared with the 10‐ (*r* = 0.478, *p* < 0.003 and 95% CI: −0.118–0.808) and 15‐s test durations (*r* = 0.397, *p* = 0.180 and 95% CI: −0.213–0.771).

**TABLE 3:** – Comparison of performance variables across three experimental conditions—10, 15 and 30 s (*n* = 13).

Exercise duration	10 s	15 s	30 s		
Performance variables	M ± SD	M ± SD	M ± SD	*p*‐value	*η* ^*2*^_*p*_
PP (W)	1123.54 ± 180.13	1098.54 ± 184.96	1086.25 ± 181.06	*^a^* *p* = 0.015	*η* ^*2*^_*p*_ = 0.30
PP (W·kg^−1^)	14.83 ± 1.62	14.34 ± 1.71	14.35 ± 1.85	*^a^* *p* = 0.02	*η* ^*2*^_*p*_ = 0.28
FI (%)	37.27 ± 7.03	45.17 ± 6.88	64.60 ± 6.15	*^a^* *p* < 0.01	*η* ^*2*^_*p*_ = 0.92
Max pedalling rate (rpm)	156.85 ± 8.42	155.54 ± 8.56	153.77 ± 8.73	*^a^* *p* < 0.01	*η* ^*2*^_*p*_ = 0.40
Mean pedalling rate (rpm)	145.31 ± 7.12	142.38 ± 7.04	132.69 ± 5.65	*^a^* *p* < 0.01	*η* ^*2*^_*p*_ = 0.92
MP (W)	892.23 ± 116.39	818.54 ± 110.95	667.77 ± 81.23	*^a^* *p* < 0.01	*η* ^*2*^_*p*_ = 0.93
MP (W·kg^−1^)	11.83 ± 1.36	10.76 ± 0.97	8.90 ± 0.99	*^a^* *p* < 0.01	*η* ^*2*^_*p*_ = 0.93
Tpeak‐3.5%	2.56 ± 1.10	2.86 ± 1.26	2.53 ± 1.80	*p* = 0.625	*η* ^*2*^_*p*_ = 0.03
Tpeak	1.52 ± 0.61	1.44 ± 0.42	1.46 ± 0.44	*p* = 0.887	*η* ^*2*^_*p*_ = 0.01
*v*La_max_ (mmol·L^−1^)	0.83 ± 0.15	0.67 ± 0.13	0.43 ± 0.06	*^a^* *p* < 0.01	*η* ^*2*^_*p*_ = 0.93

^a^ Denotes significance *p* < 0.05; kg:; kilograms; m: mean; η^*2*^_*p*_: partial eta squared; SD: standard deviation; rpm: revolutions per minute; Tpeak: time to peak power; *v*La_max_: maximum lactate accumulation rate; W: Watts and Δ: delta.

## 4 DISCUSSION

Multiple findings support our hypothesis that the application of mNIRS can be a useful tool in identifying the optimal *v*La_max_ test duration: (1) a very strong inverse relationship between SmO_2_ desaturation and *v*La_max_ kinetics, (2) a significant inverse relationship between SmO_2_ time spent at the nadir and *v*La_max_ and (3) a moderate relationship and similar time to attain the SmO_2_ nadir (8.47 ± 1.50 s) and *v*La_max_ (8.92 ± 0.77 s). Subsequently, mNIRS may provide practical applications in determining the optimal *v*La_max_ test duration tailored to the individuals SmO_2_ desaturation profile during all‐out sprint cycle ergometry.

A 15‐s test duration to determine *v*La_max_ is frequently adopted within scientific literature (Hauser et al., 2014; Quittmann et al., 2021; Yang et al., 2023). However, our previous findings (Langley et al., 2024) suggest a shorter test duration of 10 s evokes a higher *v*La_max_ more indicative of a true maximal glycolytic flux. Furthermore, our mathematical modelling of the time dependent *v*La_max_ suggests peak values are elicited at ∼9 s during all‐out cycling (Figure 3). These findings support maximal glycolytic flux peaks between 5 and 10 s during all‐out cycling as suggested by Beneke et al. (2002). Moreover, a test duration of ∼10 s to elicit the highest *v*La_max_ is supported by Mavroudi et al. (2023) findings who demonstrated a swimming distance of 25 m corresponding to a mean time of 11.75 ± 1.38s elicited the highest *v*La_max_ (0.75 ± 0.18 mmol·L^−1^·s^−1^) in elite swimmers. In accordance with our results, Mavroudi et al. (2023) demonstrated *v*La_max_ reduced the greater the test distance (35 m [17.76 ± 2.04 s]: 0.54 ± 0.18 mmol·L^−1^·s^−1^ and 50 m [26.78 ± 3.21s]: 0.49 ± 0.16 mmol·L^−1^·s^−1^), respectively. Similarly, longer test durations lead to lower peak power and lower desaturations rates, likely due to pacing strategies in play. This was also alluded to in a similar article assessing 10 versus 30s Wingate tests (Adam et al., 1999). The findings from this study and recent experimental findings (Dunst et al., 2023; Langley et al., 2024; Mavroudi et al., 2023) all support Heck et al.’s (2003) mathematical model identifying a 10‐s test period optimal to determine the highest attainable *v*La_max._

The shorter test duration of 10 s appears optimal to determine *v*La_max_ compared with longer test durations. This is due to the suppression of phosphofructokinase (PFK) activity the longer the maximal exercise duration, resultant from elevated hydrogen protons and associated with a reduction in glycolytic flux (Mader, 2003). Moreover, the high rate of ATP hydrolysis was associated with longer test durations. This shifts the cell pH to an acidic nature favouring O_2_ dissociation supporting an increase in the rate of oxidative energy metabolism (Mader, 2003).

The rapid increase in PCr dephosphorylation reflects the instantaneous energy demand of the working muscles at the onset of maximal exercise (Chung et al., 1998). The results from this study (Figure 3) demonstrate a significant correlation between SmO_2_ desaturation and the time dependent changes in PCr work, in agreement with previous research (Dunst et al., 2023; Hamaoka et al., 2007; Jones et al., 2008). Our results illustrate PCr time dependent work closely match Dunst et al.’s (2023) findings, showing the PCr work is rapid during the first 15 s and reaches a quasi‐steady state by ∼20 s, reflective of PCr work capacity. This study observed no significant differences between the SmO_2_ resaturation kinetics following the three differing sprint durations, suggesting PCr stores were either maximally depleted or similar utilisation across test durations. Excellent intraclass reliability of peak power and the maximal pedalling rate support this suggestion.

During all‐out exercise, the PCr energy supply has been shown to plateau after 10–15 s with little to no energy provided by phosphagen stores beyond this point (Barclay, 2017). Maximal ATP production can only be sustained until PCr stores decline to a maximum of half (Chung et al., 1998; Edwards et al., 1982). Subsequently, the glycolytic energy production must rapidly accelerate to compensate for the reduction in ATP production from the PCr system. Figure 3 illustrates the *v*La_max_ is already rapidly accelerating by the time 50% maximal PCr work capacity is achieved (4.5 s) corresponding to the τ *v*La_max_ (4.4 s) indicative of a significant increase in glycolytic flux by this time point. The glycolytic flux is primarily dictated by PFK activity which is activated by free ADP, AMP, Cr and P_i_, of which are produced during the rapid dephosphorylation of ATP and rephosphorylation from PCr (Mader, 2003). The energy system utilisation is closely reflected within the SmO_2_ kinetics with the intercept of the SmO_2_ desaturation τ occurring at 4.5 s closely corresponding with the *v*La_max_ τ and 50% PCr work. Additionally, the time to *v*La_max_ (8.92 ± 0.77s) and to the SmO_2_ nadir (8.47 ± 1.50s) are associated, with the greater time spent at the SmO_2_ nadir reflected in a reduction in *v*La_max_. Both Perry et al. (2024) and Jones et al. (2018) outline that an increase in SmO_2_ of a working muscle increases the muscle oxygen extraction, thus increasing the oxygen available to meet the energy demand of the activity via oxidate phosphorylation and glycolysis. In turn, the greater the energy production of the oxidative system the lower the energy demand on the anaerobic glycolytic system, reducing the production of lactate (Bartoloni et al., 2024). The reduction in *v*La_max_ also reflects a decrease in muscle pH and subsequent decreases in the maximal glycolytic flux (Mader, 2003). Consequently, the reduction in PCr stores and decline in glycolytic activity reduces the maximal force which can be produced particularly within the type II myosin heavy chain fibers (Greenhaff et al., 1994).

### 4.1 Performance data and exercise duration

While the 10‐s test duration elicited the highest *v*La_max_, it should be noted that the strongest correlation between mean power (W·kg^−1^) and *v*La_max_ was observed for the 30‐s test duration. While these correlate due to the amount of work completed, it is resultantly due to the maximum rate of glycolytic flux being reduced inverse to the exercise duration. These results agree with Mavroudi and colleagues’ (Mavroudi et al., 2023) findings who noted the 50‐m sprint which lasted 27 s had the highest correlation with swimming speed. It is important to note that the duration of the exercise is more important than the modality. The stronger correlations with the 30‐s test durations suggest the glycolytic system is the predominant energy provider for sprint durations longer than 15 s (Mader, 2003). The rate of energy produced by the PCr system decreases with exercise duration, leading to a greater percentage of the energy available being provided via glycolytic energy metabolism and utilisation (Mader, 2003; Yang et al., 2023). Despite *v*La_max_ peaking within the first 10s of exercise (Figure 3), the lower correlation with mean power (W·kg^−1^) may be attributed to the significant PCr energy contribution (Langley et al., 2024; Yang et al., 2023). Additionally, this reliance on PCr energy contribution during shorter exercise durations is in part due to the speed of muscle oxygen extraction as outlined in Table 2. The speed of oxygen extraction has been shown to be correlated with the production of energy via PCr and glycolytic energy systems (Bartoloni et al., 2024).

### 4.2 Limitations

While this study only employed high‐quality mathematical models, the validity of these models is dependent upon maximal effort to elicit both reliable performance and metabolic stress on each trial. However, it should be noted the test order was randomised, and excellent intraclass reliability was observed for peak power and rpm, indicative of maximal effort across trials.

Beyond the scope of this study, the muscle fiber typology of an individual may influence the optimal test duration of an individual. Athletes with a higher percentage of type II fibres metabolise PCr and glycogen at a quicker rate, and are associated with greater contractile force (De Luca & Contessa, 2015; Esbjornsson‐Lilljedahl et al., 1999). Subsequently, short test durations to determine *v*Lamax may be preferential for type II dominant athletes, whereas longer durations maybe more suited to type I dominant athletes. Furthermore, caution should be applied when extrapolating these findings to a female population who may express differing maximal rates of glycolysis due to lower activities of PFK, pyruvate kinase, lactate dehydrogenase, hexokinase, glycogenolysis phosphorylase, and succinic dehydrogenase (Jaworowski et al., 2002).

Additionally, it can be acknowledged that the use of the Tpeak (minus 3.5% for the alactic time period) is not the optimal method. It is observed the reliability of the Tpeak‐3.5% was moderate to good (Tpeak‐3.5% ICC: 0.52–0.881), as opposed to only poor to moderate (Tpeak ICC: 0.41–0.525) for the Tpeak (Langley & Porter, 2024). Future research should look to adopt the recently proposed method by Dunst et al. (2023) to determine the alactic time period by applying the time of fatigue‐free power assessed from force–velocity and power–velocity profiles.

Lastly, the use of the MOXY NIRS device has been designed for athlete populations rather than primarily for scientific research. Although this is the case, a paper by McManus et al. (2018) outlined that the MOXY and the PortaMon (gold standard portable NIRS device) produce similar absolute muscle oxygen saturation values. The MOXY was selected due to its versatility and practical applications over other NIRS devices. One way to reduce the margin of error within the MOXY device was to measure adiposity levels and use a muscle belly that limits device movement during exercise (McManus et al., 2018), both incorporated into the methodology used in this study.

### 4.3 Practical applications

The practical applications of these findings are as follows: (1) At a group level, the shorter test duration of 10 s should be applied when determining an athletes’ *v*La_max_. (2) Monitoring an individual's SmO_2_ desaturation profiles may help to identify an optimal test duration to determine *v*La_max_ at an individual level. (3) SmO_2_ demonstrates the ability to be used in training programme design if either the PCr ([re]saturation rate) or glycolytic stress (time desaturated) were key aims.

## 5 CONCLUSION

Our results demonstrate multiple relationships between the SmO_2_ kinetics and energy system interactions supporting our hypothesis where mNIRS can be a useful tool in identifying the optimal *v*La_max_ test duration: (1) a very strong inverse relationship between SmO_2_ desaturation and *v*Lamax kinetics, (2) a significant inverse relationship between SmO_2_ time spent at the nadir and *v*Lamax, (3) a moderate relationship between the time to attain the SmO_2_ nadir and *v*La_max_ and (4) modelled data demonstrating close correspondence between the τ SmO_2_, τ *v*La_max_ and 50% PCr work—all occurring within 0.1 s. Subsequently, monitoring the individuals’ SmO_2_ desaturation profile during all‐out sprint cycle ergometry may help to identify the optimal test duration to determine *v*La_max_, with the optimal duration likely to occur at the same point of the SmO_2_ nadir.

## AUTHORS’ CONTRIBUTIONS

MP and JL collaborated equally on all aspects of the process, from study conception, data collection, data analysis, and manuscript write‐up. MP and JL approved the final version of this manuscript for publication.

## CONFLICT OF INTEREST STATEMENT

The authors declare no conflicts of interest.
